# Monitoring and management of autoimmunity in multiple sclerosis patients treated with alemtuzumab: practical recommendations

**DOI:** 10.1007/s00415-018-8822-y

**Published:** 2018-03-10

**Authors:** Virginia Devonshire, Richard Phillips, Hilary Wass, Gerald Da Roza, Peter Senior

**Affiliations:** 1 Department of Medicine, University of British Columbia, Vancouver, BC Canada; 2 Victoria, BC Canada; 3 Division of Endocrinology and Metabolism, Department of Medicine, University of Alberta, Edmonton, AB Canada; 4 Djavad Mowafaghian Centre for Brain Health, Vancouver, BC Canada

**Keywords:** Multiple sclerosis, Autoimmunity, Alemtuzumab, Thyroid disorder, Immune thrombocytopenia, Nephropathy

## Abstract

Alemtuzumab is a humanized anti-CD52 monoclonal antibody approved in more than 65 countries for the treatment of relapsing–remitting multiple sclerosis (RRMS). Compared with subcutaneous interferon-beta-1a, alemtuzumab significantly reduced clinical disease activity and the rate of brain volume loss, and improved disability outcomes in patients with active RRMS who were either treatment naive (CARE-MS I study) or who had an inadequate response (≥ 1 relapse after ≥ 6 months of treatment) to prior therapy (CARE-MS II study). Adverse events (AEs) associated with alemtuzumab include infusion-associated reactions, infections, and autoimmunity. The most commonly reported autoimmune AEs observed with alemtuzumab involve the thyroid gland, followed by immune thrombocytopenia and nephropathies. A monitoring program was designed and implemented to facilitate the early detection of autoimmune events to ensure timely and adequate management. The aim of this article is to provide physicians (including neurologists, general practitioners, endocrinologists, hematologists, and nephrologists who may be less familiar with the symptoms and treatment of autoimmune events), with practical real-world recommendations for the monitoring and management of autoimmunity associated with alemtuzumab treatment.

## Introduction

Multiple sclerosis (MS) is a chronic autoimmune demyelinating disorder of the central nervous system. The clinical course of MS is variable, usually beginning with recurrent and reversible episodes of neurologic disability termed relapses; this phase of the disease is termed relapsing–remitting MS (RRMS). Relapses can occur months or even years apart [1]. After a number of years, the majority of RRMS patients enter a second disease phase [called secondary progressive MS (SPMS)], which is characterized by continuous, irreversible neurologic decline. Transition to this phase of MS is ominous, as therapies are not yet available to treat the cognitive and physical decline that occurs. Prevention of this transition to SPMS is, therefore, a major therapeutic goal of MS treatment.

Alemtuzumab (LEMTRADA^®^; Sanofi Genzyme, Cambridge, MA, USA) is a humanized anti-CD52 (cluster of differentiation 52) monoclonal antibody approved in more than 65 countries for the treatment of RRMS. Alemtuzumab treatment results in the selective depletion and repopulation of circulating CD52-expressing T and B lymphocytes. Following depletion, a distinct pattern of T- and B-cell repopulation begins within weeks, including a relative increase of regulatory T cells and a decrease in proinflammatory cytokines [2, 3]. These pharmacological effects potentially lead to a rebalancing of the immune system and may underlie its durable clinical effects in the absence of continuous treatment. Alemtuzumab is administered as 2 annual courses of 12 mg/day intravenously, with the first course given over 5 consecutive days at treatment initiation, and the second course 12 months later over 3 consecutive days.

The efficacy and safety of alemtuzumab have been evaluated in treatment-naive RRMS patients [phase 2 CAMMS223 study (ClinicalTrials.gov identifier: NCT00050778) and phase 3 CARE-MS I (NCT00530348)] and in RRMS patients who had an inadequate response (≥ 1 relapse after ≥ 6 months of treatment) to prior therapy [CARE-MS II study (ClinicalTrials.gov identifier: NCT00548405)]. Alemtuzumab significantly reduced the rate of clinical disease worsening over 36 months in the phase 2 CAMMS223 study [4]. In the phase 3 CARE-MS trials, alemtuzumab demonstrated significantly greater improvements in disease activity over 2 years versus subcutaneous interferon beta-1a (SC IFNB-1a) administered three times per week [5, 6]. In both CARE-MS I and II studies, alemtuzumab significantly reduced the frequency of relapses over 2 years compared with SC IFNB-1a, significantly improved MRI outcomes, including gadolinium-enhancing lesions and new or enlarging T2 hyperintense lesions, and significantly reduced the rate of brain volume loss [5, 6]. Patients treated with alemtuzumab in CARE-MS II also showed a significantly reduced rate of 6-month confirmed disability worsening (CDW) versus SC IFNB-1a, with patients from the CARE-MS I study showing a nonsignificant 30% reduction in CDW. In addition, in CARE-MS II, patients treated with alemtuzumab were more than twice as likely as patients treated with SC IFNB-1a to experience 6-month confirmed disability improvement (hazard ratio 2.57, *p *= 0.0002) [6].

Patients who completed the CAMMS223 and CARE-MS trials could enter an open-label extension study to allow evaluation of long-term alemtuzumab efficacy and safety. Alemtuzumab demonstrated durable efficacy on clinical and MRI outcomes over 10 years in patients who were originally enrolled in CAMMS223 [7] as well as durable efficacy over 6 years (2 years of core study plus 4 years of extension) in the absence of continuous treatment in patients who were originally enrolled in the CARE-MS studies [7]. Annualized relapse rate remained low, most patients were free of CDW, and some patients also continued to achieve improvements in disability over 6 years [7]. These results were achieved with a high retention rate over the 6-year period (82% of patients who enrolled in the CARE-MS studies remained on study through Year 6), and with the majority (56%) of patients who entered the CARE-MS extension study receiving no additional treatment for their MS (no alemtuzumab retreatment nor use of another disease-modifying treatment).

All disease-modifying therapies for MS have side effects. The most common adverse events (AEs) associated with alemtuzumab treatment are mild to moderate infusion-associated reactions, infections (mostly nonserious), and autoimmune AEs [7–11]. Through 6 years of follow-up, AEs involving the thyroid gland were the most frequently reported autoimmune AEs, observed in 42% of patients treated with alemtuzumab (which included both hyperthyroidism and hypothyroidism) [11]. Other autoimmune AEs observed in patients treated with alemtuzumab were low platelet counts [(immune thrombocytopenia (ITP)] and nephropathies.

Following a fatality associated with ITP in the phase 2 CAMMS223 trial, a safety monitoring program was implemented to facilitate the early detection and management of autoimmune events. The program includes physician and patient education about the possible signs and symptoms of autoimmune events, as well as monthly monitoring for renal and hematologic events and quarterly monitoring of thyroid function [throughout treatment and for 48 months after the last alemtuzumab course (second or subsequent course)].

The cause of autoimmunity in alemtuzumab-treated patients is not well understood, but may be related to the pattern of T- and B-cell depletion and repopulation following alemtuzumab treatment. The repopulation of T cells may occur through two pathways: (1) thymopoiesis and (2) homeostatic proliferation of cells that have escaped depletion. Autoimmune disease may be more likely when homeostatic proliferation predominates over thymic reconstitution [12]. Another hypothesis is that the depletion followed by the rapid repopulation of B cells may be a factor in the development of autoimmunity with alemtuzumab, particularly in individuals with a genetic predisposition for autoimmunity [13]. However, evidence to support these hypotheses is lacking, and additional studies are needed to elucidate the cause of autoimmunity in alemtuzumab-treated patients.

Although the safety monitoring program was designed and implemented to facilitate the timely detection of autoimmune events related to alemtuzumab, and despite some recommendations for the management of thyroid-related events [14], there remains a paucity of published practical recommendations on how to monitor and manage these events if detected in clinical practice. In particular, there continues to be uncertainty regarding when, and which, patients with renal and hematologic events may require referral to another specialist.

Based on the published literature and the authors’ own real-world experiences, this article offers practical real-world recommendations for the monitoring, management, and referral of MS patients with possible autoimmune AEs (thyroid events, ITP, and nephropathies) associated with alemtuzumab treatment.

## Recommendations for the monitoring and management of autoimmunity in alemtuzumab-treated patients

### Thyroid events

#### Background on thyroid events

**Fig. 1:**
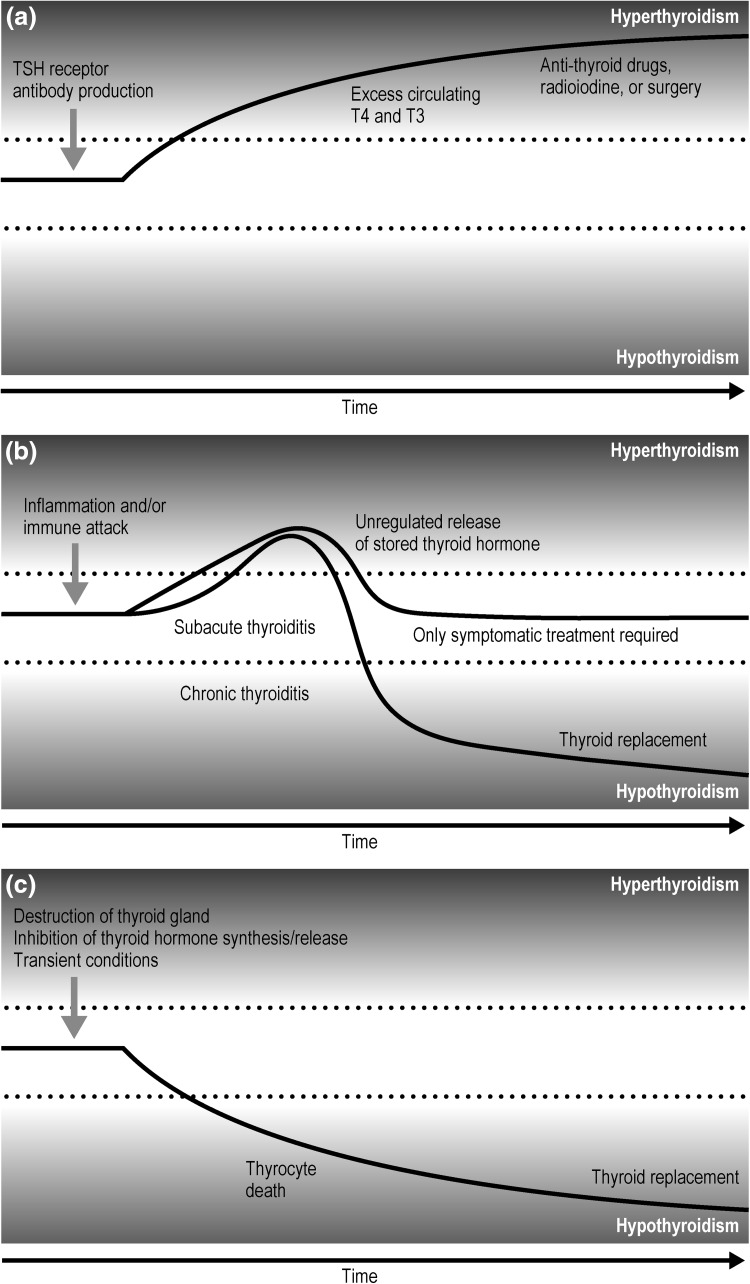
Autoimmune thyroid disease. **a** Hyperthyroidism: Graves’ disease/thyrotoxicosis; **b** painless thyroiditis; and **c** hypothyroidism. *T3* triiodothyronine, *T4* thyroxine, *TSH* thyroid-stimulating hormone

There is a spectrum of autoimmune thyroid disorders ranging from Hashimoto’s disease, in which damage to the thyroid gland may result in permanent hypothyroidism, to Graves’ disease, in which antibodies against the thyroid-stimulating hormone (TSH) receptor stimulate excessive function of the gland, resulting in hyperthyroidism. Graves’ disease is the most common form of hyperthyroidism [thyrotoxicosis: excessive amounts of circulating thyroid hormones, thyroxine (T4) and triiodothyronine (T3)] (Fig. 1a), and usually requires treatment with anti-thyroid drugs, radioiodine, or surgery. A transient, and less common, cause of hyperthyroidism due to autoimmunity is “painless thyroiditis”, in which inflammation damages the thyroid, resulting in unregulated release of preformed thyroid hormone into the circulation (Fig. 1b). Only symptomatic treatment is required, and when these stores are depleted, the hyperthyroidism usually resolves. However, in some cases, chronic thyroiditis and hypothyroidism may result, which can become permanent and necessitate thyroid hormone replacement. Notably, TSH-receptor antibodies are not involved in the development of painless thyroiditis, so this test can be used to differentiate painless thyroiditis from Graves’ hyperthyroidism.

Hypothyroidism (a state of low circulating thyroid hormone) is routinely managed in primary care. It can be associated with a number of conditions including destruction of the thyroid gland (surgical removal, irradiation, autoimmune disease, idiopathic atrophy, or infiltrative process), inhibition of thyroid hormone synthesis/release (iodine deficiency, excess iodide, certain medications [IFN-alpha, lithium], inherited enzyme defects), and transient conditions (therapeutic radioiodine, postpartum, or thyroiditis) (Fig. 1c) [15]. These conditions can result in thyrocyte death and lead to hypothyroidism, which can be treated with thyroid replacement. In areas of the world with sufficient dietary iodine, hypothyroidism is most commonly caused by Hashimoto’s thyroiditis (chronic lymphocytic thyroiditis) [16], which is characterized by infiltration of the thyroid gland with T lymphocytes and autoantibodies against specific thyroid antigens such as thyroid peroxidase or thyroglobulin.

Patients with MS already have a significantly greater incidence of Graves’ disease compared with the general population (3.1 versus 0.4%, respectively; *p* = 0.002), although the prevalence of Hashimoto’s thyroiditis was not significantly different between the two populations (5.5 and 2.2%, respectively, *p *= 0.097) [17].

Thyroid abnormalities are frequently detected incidentally on routine biochemical testing in relatively asymptomatic patients. Symptoms of hypothyroidism are often nonspecific, may develop slowly over time, and can have a long preclinical phase [14]. Symptoms of hyperthyroidism tend to be more pronounced, except in very early or mild cases, and are frequently characterized by symptoms of increased sympathetic nervous system activity [18].

In the large, 10-year, phase 2 study of alemtuzumab in treatment-naive patients with RRMS (CAMMS223), the rate of thyroid AEs peaked in Year 3 (17%) following the first course, with serious thyroid events occurring in fewer than 4% of patients during each year through Year 10 [10]. This was similar to rates of thyroid events observed in the phase 3 CARE-MS studies, which also peaked 3 years following the first course of alemtuzumab (16%) and were primarily mild or moderate in severity [11]. Through 6 years in the phase 3 studies, 42% of patients experienced a thyroid AE, including hyperthyroidism (including Graves’ disease), hypothyroidism, thyroiditis, and goiter. Few serious thyroid AEs were reported (incidence peaked at 3.1% in Year 3 and subsequently declined), and no deaths occurred as a result of thyroid events.

Data from the phase 2 CAMMS223 study suggest that alemtuzumab-related thyroid dysfunction has some atypical features compared with autoimmune thyroid diseases in the general population. Alemtuzumab-associated Graves’ disease was more likely to spontaneously revert to normal, or convert to hypothyroidism, whereas alemtuzumab-associated primary hypothyroidism is much more likely to be mediated by TSH-receptor-blocking antibodies than in the general population [19].

In the CARE-MS studies, 20.7% of patients who experienced their first thyroid event through 6 years received no treatment. In those patients who received treatment, most (79.0%) were managed with conventional oral medications, 9.0% requiring iodine ablation and 8.5% undergoing thyroidectomy [11].

#### Recommendations for monitoring and management of thyroid events in alemtuzumab-treated patients

**Fig. 2:**
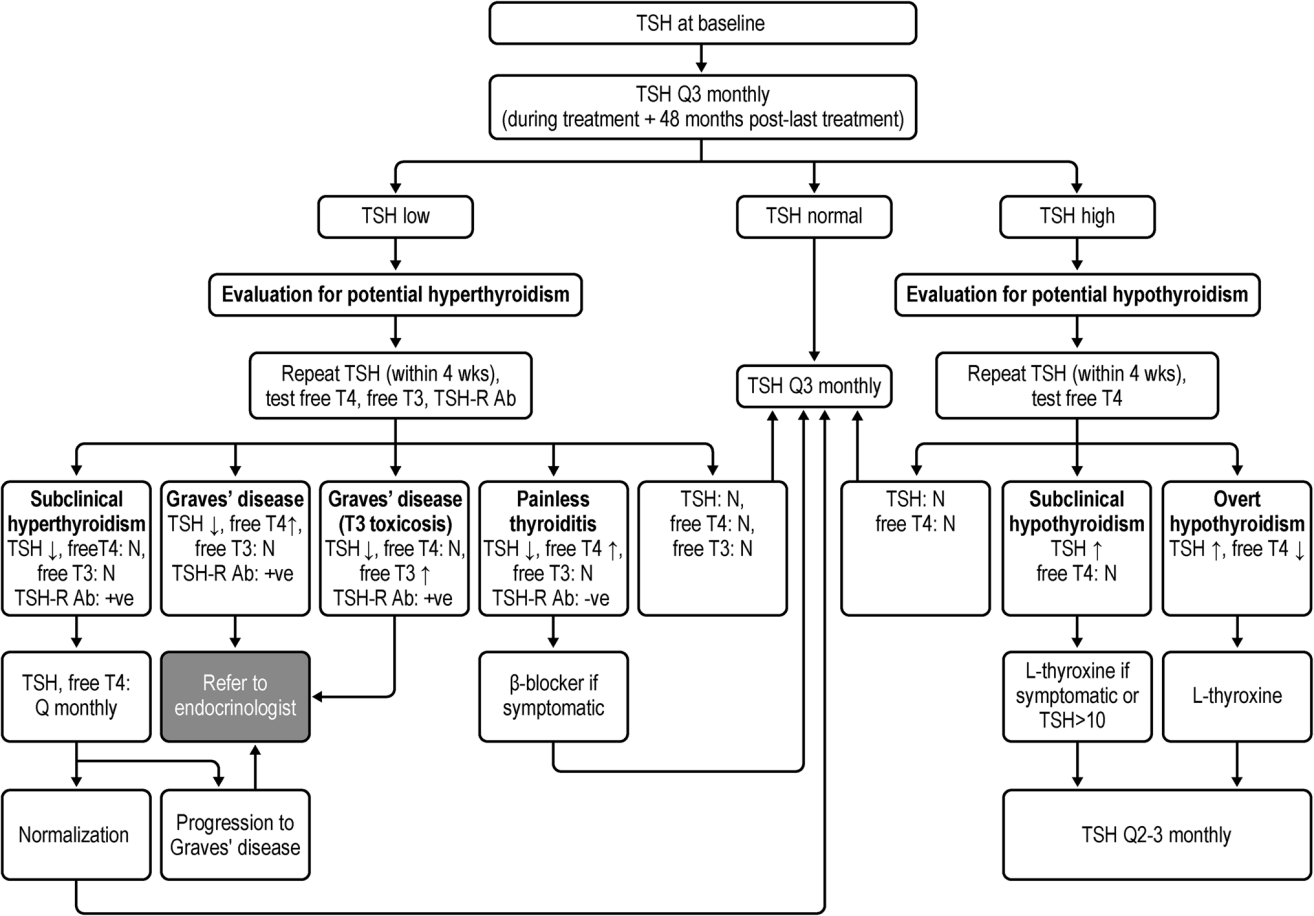
Practical recommendations for the monitoring of thyroid function in patients treated with alemtuzumab. *N* normal, *Q3 monthly* every 3 months, *T3* triiodothyronine, *T4* thyroxine, *TSH* thyroid-stimulating hormone (normal: 0.4–4 mU/L), *TSH*-*R Ab* TSH-receptor antibody. Note: If patient becomes pregnant, establish a new TSH baseline and/or consider monitoring more frequently

A proposed monitoring algorithm for thyroid events in alemtuzumab-treated patients is shown in Fig. 2. TSH measurements should be performed at baseline and every 3 months for 48 months following the last course (second or subsequent course) (Table 1; Fig. 2). Normal ranges for TSH values vary to a small degree between different assays, but generally range from 0.4 to 4.0 mU/L. If the TSH levels are abnormal, or should patients develop symptoms of hyper- or hypothyroidism (Table 2), further laboratory investigation (including free T4, free T3, and TSH-receptor antibody testing) along with an assessment of thyroid symptoms is required. The patient should be evaluated for potential hyperthyroidism if TSH levels are less than the normal range and for potential hypothyroidism if the levels are more than the normal range.

**Table 1:** Routine monitoring for autoimmune events in alemtuzumab-treated patients

Test	Timing
Thyroid function	
TSH	□ Prior to initial treatment □ Every 3 months for 48 months following last course (second or subsequent course)
Hematology	
Platelet count	□ Prior to initial treatment □ Monthly for 48 months following last course (second or subsequent course)
Kidney function	
Creatinine	□ Prior to initial treatment □ Monthly for 48 months following last course (second or subsequent course)
Urine analysis	□ Prior to initial treatment □ Monthly for 48 months following last course (second or subsequent course)

**Table 2:** Signs and symptoms of autoimmune events

Autoimmune condition	Symptoms	Signs
Thyroid events [15, 16]		
Hypothyroidism	Fatigue, weight gain, cold intolerance, dry skin and hair, mood disorder, and constipation	Hypothyroid facies, goiter, and delayed relaxation phase of tendon reflex
Hyperthyroidism	Fatigue, weight loss, heat intolerance, shaky hands, muscle weakness, insomnia, anxiety, palpitations, and increased frequency of bowel movements	Tremor, tachycardia, upper lid retraction, proptosis (specific for Graves’ disease), goiter, and hyperreflexia
Immune thrombocytopenia [28]	Bruising and onset or exacerbation of heavy menstrual bleeding	Mucosal bleeding (oral/gastrointestinal), and life-threatening hemorrhages (rare)
Nephropathy [34]	Fatigue, dizziness and trouble concentrating; loss of appetite; nausea; metallic taste; sleep disturbance; nocturnal muscle cramping; and changes in urination	Swollen feet and ankles, itchy skin, and puffiness around the eyes; gross hematuria

#### Evaluation for potential hyperthyroidism

If the TSH levels are less than the normal range (< 0.4 mU/L), the TSH testing should be repeated within 4 weeks. Free T4 (normal range, 0.7–1.9 ng/dL), free T3 (normal range, 230–619 pg/dL), and TSH-receptor antibody levels (normal range, ≤ 1.75 IU/L) should also be assessed, with tests of free T4 and/or free T3 repeated every 4–6 weeks until levels return to normal, or treatment/referral to a specialist is indicated. Slightly low TSH levels (0.2–0.4 mU/L) with normal free T4 and free T3 levels are consistent with subclinical hyperthyroidism, and TSH can be monitored. If the patient is symptomatic with palpitations or tremor or has tachycardia, treatment with a beta-blocker may be initiated immediately (while waiting for results). Often larger doses are required for treatment of hyperthyroidism (e.g., propranolol, 80 mg daily) than might be used for heart disease or hypertension.

Overt hyperthyroidism is suspected if TSH is suppressed with elevated free T4 and/or free T3 (Fig. 2). If the patient has a positive TSH-receptor antibody titer, then a diagnosis of Graves’ disease is confirmed, whereas a negative result suggests a diagnosis of painless thyroiditis. An alternative method of distinguishing Graves’ disease from painless thyroiditis is with a nuclear medicine thyroid radioiodine uptake test, although the use of TSH-receptor antibody in place of thyroid nuclear scans is recommended based on lower cost and a shorter turnaround time [20]. Overt hyperthyroidism on the basis of Graves’ disease should prompt a referral to an endocrinologist for management, which may include anti-thyroid medications, radioiodine ablation, or thyroidectomy [21]. Painless thyroiditis or subclinical hyperthyroidism may be followed by monthly TSH and free T4 monitoring until progression to overt hyperthyroidism and/or spontaneous normalization of results, whereupon resuming TSH testing every 3 months can continue.

#### Evaluation for potential hypothyroidism

An elevated TSH (> 4.0 mU/L) should prompt assessment of hypothyroid symptoms, with a repeat TSH measurement and free T4 determination within 4 weeks; levels should be assessed every 4–6 weeks until they return to normal or treatment is indicated. Subclinical hypothyroidism is characterized by an elevated TSH with normal free T4 and is frequently asymptomatic. TSH levels of 5–10 mU/L and free T4 levels less than the reference range are consistent with overt hypothyroidism; treatment with l-thyroxine may be considered, especially if the patient is symptomatic. For asymptomatic patients with TSH levels ≥ 10 mU/L, treatment with l-thyroxine may be considered and is recommended for symptomatic patients with TSH higher than the reference range. If treatment with l-thyroxine is initiated, the TSH testing should be repeated every 4–6 weeks and the dose titrated to maintain the TSH level within the reference range.

Some patients with MS may, in theory, take large doses of biotin to prevent progression of disability. It has recently been noted that supraphysiological doses of biotin may interfere with a multitude of immunoassays including TSH, free T4, free T3, and TSH-receptor antibodies, mimicking the biochemistry of patients with Graves’ hyperthyroidism [22, 23]. Thus, it is imperative to inquire about biotin use in MS patients with abnormal thyroid function test results. If abnormal test results occur while patients are taking high-dose biotin supplements, the biotin should be withheld for 2–3 days and the test repeated.

### ITP

#### Background on ITP

ITP is an immune-mediated disorder resulting in a decrease in platelet count (platelet count < 100 × 10^9^/L) that can present as a self-limiting (< 3 months) or chronic (lasting ≥ 12 months) condition [24]. Acute ITP occurs mainly in children and often resolves without treatment. ITP in adults is typically chronic, with annual incidence ranging from 1.6 to 3.9 per 100,000 patient-years, and is often irreversible [25, 26]. ITP can have a variety of causes including systemic disease, infection, medications, and primary hematologic disorders [27].

The symptoms of drug-induced ITP can be highly variable (see Table 2) and may be diagnosed on the basis of routine laboratory screening if ITP is mild [27, 28]. Easy bruising is the most common symptom; other common symptoms include mucocutaneous bleeding, ecchymosis, and heavy menstrual bleeding. Petechiae are uncommon and are usually observed on the lower limbs of patients with a platelet count < 20 × 10^9^/L (and often < 10 × 10^9^/L).

Alemtuzumab is associated with a unique form of ITP. In adults, ITP may be chronic and irreversible, whereas ITP in alemtuzumab-treated patients is characterized by delayed onset, responsiveness to conventional therapies, and prolonged remission following treatment [29]. The overall incidence of ITP in the alemtuzumab clinical development program was 2.3%, with most cases resolving with first- or second-line therapy [30]. The mean time from last alemtuzumab dose to ITP onset was 17 months (range 1–44 months), and ITP onset was ≤ 3 years from the time of the last alemtuzumab dose in all cases except one. No further fatalities associated with ITP have been reported with alemtuzumab following the index case [11].

#### Recommendations for monitoring and management of ITP in alemtuzumab-treated patients

**Fig. 3:**
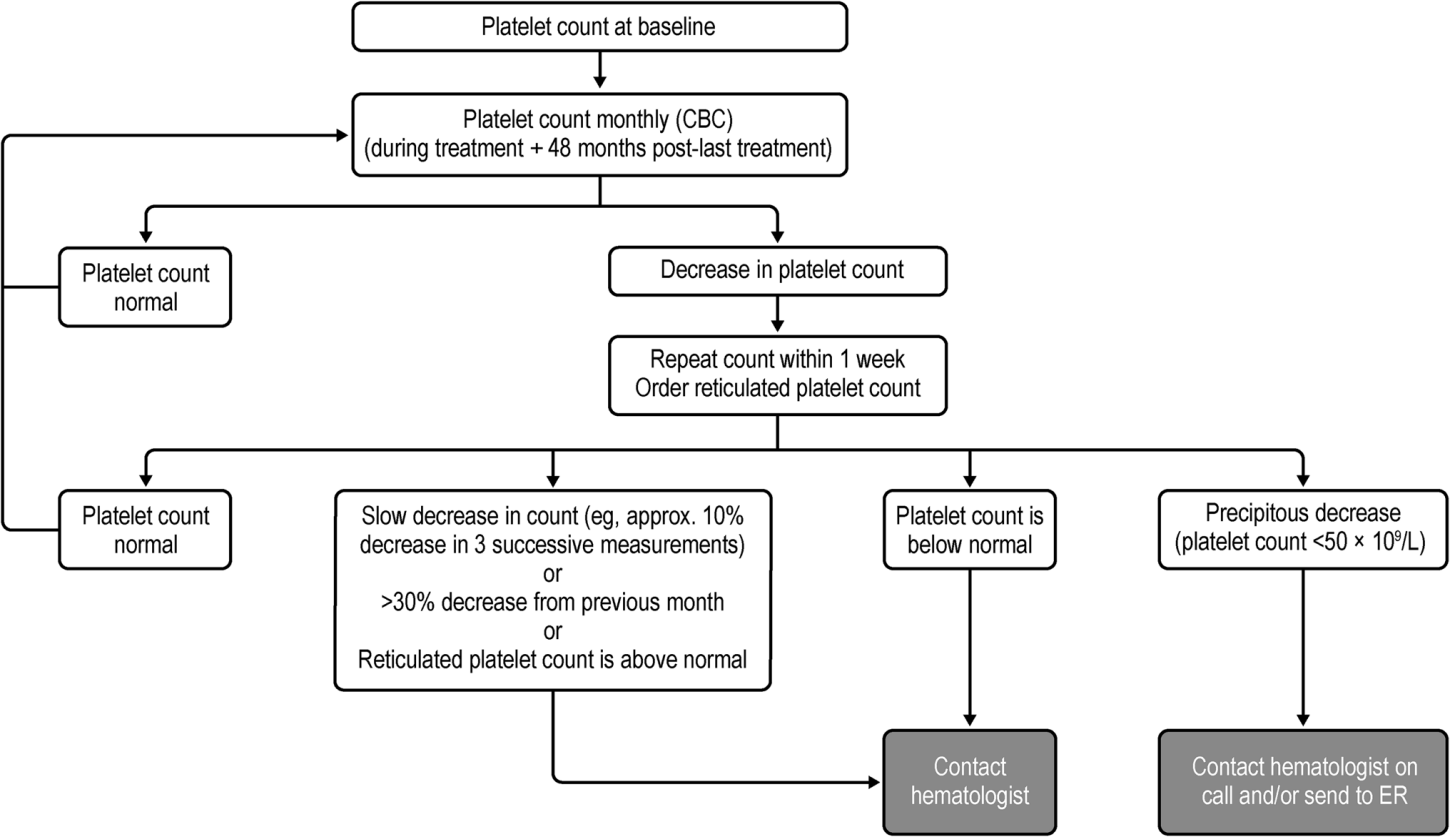
Practical recommendations for the monitoring of platelet counts in patients treated with alemtuzumab. Normal platelet count = 150–400 platelets/L. *CBC* complete blood count. Normal reticulated platelet count percentage: 3–20% and absolute reticulated platelet count: 17 ± 6.6 (10^3^/µL). Note: Platelet count can be highly variable

A proposed monitoring algorithm for alemtuzumab-related ITP is shown in Fig. 3. Platelet count should be performed before the initial course of alemtuzumab followed by monthly testing, which should be continued until 48 months after the last course (second or subsequent course; Table 1).

If a patient is thrombocytopenic (platelet counts < 100 × 10^9^/L), the test should be repeated within 1 week along with a reticulated platelet count and if:

i. there is a steep decrease in platelet count (< 50 × 10^9^/L), the patient should be evaluated urgently by a hematologist or sent to the emergency room, or
ii. there is new onset of bleeding, a slow decrease in platelet count, a > 0% decrease from previous month, or if reticulated platelet count is elevated above normal, the patient should be referred to a hematologist.

If the platelet count is ≤ 30 × 10^9^/L, treatment with corticosteroids or intravenous immunoglobulin may be required. Patients with persistent low platelet counts < 20–30 × 10^9^/L despite steroid or immunoglobulin treatment may be treated with rituximab, thrombopoietin-receptor agonists, or splenectomy (depending on specific treatment protocols) [25, 28]. Patients with refractory counts ≤ 30 × 10^9^/L may be treated with azathioprine, cyclosporine, cyclophosphamide, dapsone, or mycophenolate mofetil, although the decision to initiate third-line therapy depends on clinical circumstances and individual patient factors. Any new onset of bleeding should be assessed, especially if it occurs between regular counts; acute onset of bleeding requires an immediate platelet count and/or trip to the emergency room.

### Autoimmune nephropathy

#### Background on nephropathy

Nephropathy encompasses anti-glomerular basement membrane (anti-GBM) disease, membranous nephropathy, and tubulointerstitial nephritis. Anti-GBM disease affects the glomeruli, with immunofluorescence showing immunoglobulin deposition along the GBM. Anti-GBM is uncommon with an estimated incidence of one case per 2 million per year in the European Caucasian populations [31]. The onset is usually abrupt and often rapidly progressive. The severity of the resultant clinical syndrome ranges from mild or no renal involvement to rapidly progressive glomerulonephritis [32]. Signs and symptoms of nephropathy are often nonspecific (see Table 2) [33].

In the CARE-MS studies, two nephropathy events were reported as autoimmune AEs over 6 years (incidence: 0.2%), both within the 48-month monitoring period [11]. One case of membranous glomerulonephritis was diagnosed in Year 2 by kidney biopsy. Another case of membranous glomerulonephritis with weak seropositivity for anti-GBM autoantibodies was observed in Year 3. Both patients responded to treatment and renal function recovered. An additional case of anti-GBM disease was reported in the phase 2 CAMMS223 trial 40 months after the second course of alemtuzumab [34]. The patient responded to treatment and renal function was preserved.

#### Recommendations for monitoring and management of nephropathy in alemtuzumab-treated patients

**Fig. 4:**
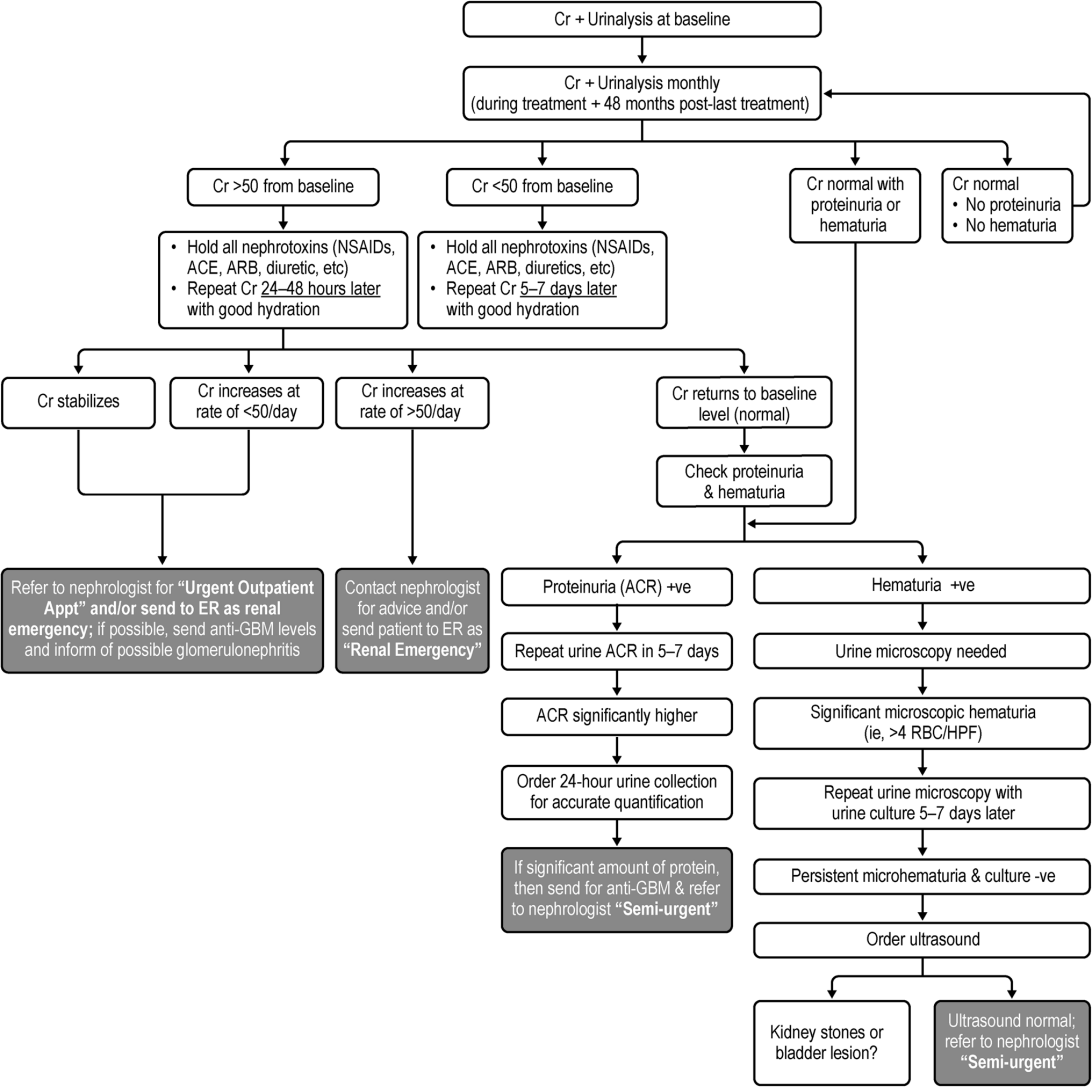
Practical recommendations for the monitoring of kidney function in patients treated with alemtuzumab. *ACE* angiotensin-converting enzyme, *ACR* albumin/creatinine ratio, *ARB* angiotensin-receptor blocker, *Cr* creatinine, *ER* emergency room, *GBM* glomerular basement membrane, *HPF* high-power field, *NSAIDs* nonsteroidal anti-inflammatory drugs, *RBC* red blood cell

A proposed monitoring algorithm for alemtuzumab-related nephropathy is shown in Fig. 4. Routine creatinine testing should be performed before treatment followed by monthly testing during treatment and continued until 48 months after the last course (second or subsequent course) (Table 1).

If the creatinine levels rise ≥ 50 µmol/L higher than baseline, all potential nephrotoxic agents should be withheld (e.g., nonsteroidal anti-inflammatory drugs, angiotensin-converting enzyme inhibitors, angiotensin-receptor blockers, diuretics), the patient should be kept hydrated, and the creatinine test should be repeated 24–48 h later. If upon retesting, the creatinine levels continue to increase at a rate of:

(i) > 50 µmol/L/day, the nephrologist should be contacted immediately for advice or the patient should be sent to hospital as a renal emergency for further investigation or,
(ii) < 50 µmol/L/day, the patient should be referred to a nephrologist for an urgent outpatient appointment (along with anti-GBM levels if possible) and the nephrologist should be informed of potential glomerulonephritis.

If the creatinine levels normalize, the patient should be tested for proteinuria and hematuria, and if the patient presents with:

(i) Positive proteinuria (albumin/creatinine ratio), the urine albumin/creatinine ratio should be repeated in 5–7 days and, if elevated, a 24-h urine collection should be ordered. If significant amounts of protein are present on 24-h collection, the patient should be sent for testing of anti-GBM levels and referred to a nephrologist as semi-urgent.
(ii) Positive hematuria, urine microscopy should be performed and, if significant (> 4 RBC/hpf), the urine microscopy should be retested 5–7 days later. If the patient has persistent microhematuria and a negative culture, an ultrasound is indicated. If the ultrasound is normal, the patient should be referred to a nephrologist as semi-urgent.

If creatinine levels change by < 50 µmol/L higher than baseline, the patient should be kept hydrated, all potential nephrotoxic agents should be withheld (e.g., nonsteroidal anti-inflammatory drugs, angiotensin-converting enzyme inhibitors, angiotensin-receptor blockers, diuretics), and the creatinine test should be repeated 5–7 days later. Any significant change in creatinine level from baseline, even if it stabilizes, should warrant a nephrology referral.

In patients with anti-GBM disease, the focus should be on removing the circulating autoantibodies and preventing any further ongoing autoimmune activity with plasma exchange, steroids, and cyclophosphamide [33]. If a patient develops end-stage renal disease, transplantation is possible once the autoantibodies become undetectable.

## Discussion

Alemtuzumab treatment can provide significant benefits for patients with RRMS, having been shown to significantly reduce relapses, CDW, and brain volume loss compared with SC IFNB-1a. The proportion of patients showing disability improvement was also more favorable in patients receiving alemtuzumab compared with SC IFNB-1a. These benefits were sustained for at least 6 years in the absence of continuous treatment and, notably, most patients did not receive additional alemtuzumab treatment or another disease-modifying therapy during the follow-up period.

Although the progression of MS is not entirely predictable, the consequences associated with not managing MS can be significant and can include more frequent relapses, progression of disability, and conversion to SPMS [35, 36]. Initiating treatment with alemtuzumab improves clinical outcomes and reduces the rate of conversion of RRMS to SPMS with a manageable safety profile [5, 6, 8–10, 37–40]. Alemtuzumab has also been shown to significantly improve physical, mental, and emotional quality of life, and the 6-year phase 3 CARE-MS extension efficacy and safety data are consistent with the 10-year phase 2 data [41, 42].

Treatment with alemtuzumab is accompanied by an increased risk for autoimmunity that most frequently involves the thyroid gland. These autoimmune events are predictable and identifiable, and are manageable with early detection and intervention. Since the implementation of the autoimmunity monitoring program, no deaths related to autoimmunity in alemtuzumab-treated patients have occurred. Furthermore, the half-life of alemtuzumab is approximately 2 weeks and serum concentrations are generally undetectable within approximately 30 days following each treatment course [43]. As most autoimmune events occur in the timeframe of months to years after infusion, these events are occurring in the absence of alemtuzumab, although they may potentially be related to the distinct pattern of T- and B-cell depletion and repopulation that occurs following alemtuzumab treatment. Additional studies are needed to definitively elucidate the cause of autoimmunity in alemtuzumab-treated patients.

Autoimmune events can have serious consequences, and patients receiving treatment with alemtuzumab will require close monitoring to mitigate risk. Should autoimmunity develop, early and appropriate intervention can lead to favorable outcomes. The recommendations detailed here will help provide healthcare professionals with a thorough understanding of the presentation of autoimmune events in alemtuzumab-treated patients to identify them in a timely manner and to initiate appropriate treatment.
